# Pirfenidone improves survival in IPF: results from a real-life study

**DOI:** 10.1186/s12890-018-0736-z

**Published:** 2018-11-23

**Authors:** George A. Margaritopoulos, Athina Trachalaki, Athol U. Wells, Eirini Vasarmidi, Eleni Bibaki, George Papastratigakis, Stathis Detorakis, Nikos Tzanakis, Katerina M. Antoniou

**Affiliations:** 1 Respiratory Medicine Department, University Hospital of Heraklion, Crete, Greece; 2 Interstitial Lung Disease Unit, Royal Brompton Hospital, London, UK; 3 Radiology Department, University Hospital of Heraklion, Crete, Greece

**Keywords:** IPF, Survival, Pirfenidone, Real-life

## Abstract

**Background:**

Pirfenidone is an antifibrotic compound approved for the treatment of idiopathic pulmonary fibrosis (IPF). We present our real-world experience in terms of Pirfenidone’s effect on mortality and adverse events profile outside the restrictions of a clinical trial.

**Methods:**

This is a retrospective observational intention to treat study of 82 consecutive IPF patients (UHH cohort).

**Results:**

We observed a high 3-years survival rate of 73% without excluding patients who discontinued treatment for different reasons. The survival was compared to the survival of an IPF cohort from a tertiary referral center (RBH cohort). After exclusion of severe cases (DLco< 30%), in unadjusted analysis, the survival in the UHH cohort was better than in the RBH cohort (HR:0.32, 95% CI: 0.19–0.53, *p* < 0.0001). After adjustment for age, gender and FVC, the survival remained higher in the UHH cohort (HR:0.28, 95% CI: 0.16–0.48, *p* < 0.0001). We observed a similar safety profile compared to previously published data and a lower rate of drug discontinuation due to photosensitivity reactions. Conclusion: Pirfenidone provides a survival benefit in a real-life IPF cohort compared to previously used medications. Counselling patients and proactively managing possible adverse effects can reduce the necessity to discontinue pirfenidone.

## Background

Idiopathic pulmonary fibrosis (IPF) is a rare disease of unknown etiology, characterized by progressive and irreversible fibrosis of the interstitium of the lung [1, 2]. The prevalence of IPF in Europe and North America has been estimated to be from 3 to 9 cases per 100,000 [3].

Pirfenidone is an orally administered drug with antifibrotic, anti-inflammatory and antioxidant effects that was approved in Europe in 2011 and in USA in 2014 for the treatment of IPF [4]. Together with nintedanib, the second antifibrotic compound, pirfenidone has received the label of “conditional recommendation for IPF treatment” in the recent update of the ATS/ERS/JRS/ALAT 2015 statement [5].

Three multinational, randomised, placebo-controlled phase III trials showed that pirfenidone can reduce the rate of IPF progression by 50% on average in 1 year as judged by serial changes in forced vital capacity (FVC) [6]. These trials were not powered to explore the effect of pirfenidone on mortality. However, a prespecified pooled analysis showed that pirfenidone reduced the risk of death at 1 year by 48% and the risk of treatment-emergent death due to IPF at 1 year by 68%. More recently, a pooled analysis of these trials and a meta-analysis including also a Japanese phase 2 trial, SP2 (trial duration 9 months), and a Japanese phase 3 trial, SP3 (trial duration 52 weeks) confirmed that pirfenidone is associated with a reduced risk of death compared to placebo and moreover the survival benefit of pirfenidone extended beyond 52 weeks and seemed to persist at 120 weeks [7].

The use of this type of analysis to study the effect of pirfenidone on mortality is justified by the fact that it is impossible to design a single trial, powered enough to provide a significant effect on mortality. Clinical trials adequate for evaluation of mortality as an end-point should include an extremely high number of patients and would require a much longer follow-up time in order to reach statistical significance [8].

In the recently published RECAP study, an open-label extension study evaluating the long-term safety of pirfenidone in patients with IPF who completed the phase III trials, the median on-treatment survival from the first dose of 2403 mg/day pirfenidone was 77.2 months confirming a survival benefit compared to historical data [9, 10]. However, this result should be interpreted cautiously because only patients who remained under observation for the whole study period were included in the survival analysis whereas patients who withdrew at different time-points for different reasons were excluded. This might have shifted the results towards a better survival. Furthermore, there is a doubt regarding the applicability of these observations obtained from pharmaceutical cohorts to the general IPF population. Patients with advanced disease and comorbidities were excluded. It is well recognised that baseline disease severity and presence of comorbidities have a significant impact on survival [11, 12]. Therefore, longitudinal real-world data from cohorts in which patients with advanced disease and comorbidities are included, are needed to shed light to the mortality benefit of pirfenidone in the general IPF population.

The aim of our study was to investigate the efficacy of Pirfenidone in newly diagnosed IPF patients presented in a referral centre. To strengthen our results, we compared the survival in our cohort with the survival in a historic IPF cohort studied in the pre-pirfenidone era [13]. We also investigated the safety profile of pirfenidone in comparison with the data obtained from the clinical trials.

## Methods

This is a retrospective observational study aiming to assess the efficacy and safety of pirfenidone in IPF patients**.** Ninety consecutive IPF patients referred to the Interstitial Lung Diseases outpatient clinic of the University Hospital of Heraklion from July 2011 (when the manufacturer funded Named Patient Programme was initiated) to December 2016 were included in the study (herein referred to as the UHH cohort). All the patients were treatment naïve and completed at least 3 months of treatment with pirfenidone (2403 mg/day). We excluded 8 patients because the gap between diagnosis and treatment introduction was greater than 12 months and therefore the final UHH cohort included 82 patients. The patients remained under follow-up at UHH and their follow-up was complete at the specified cut-off date of 3 years. The diagnosis of IPF was based on ATS/ERS/JRS/ALAT criteria [10]. In case of absence of a definite usual interstitial pneumonia (UIP) pattern on high resolution computed tomography (HRCT) and of inability to obtain a biopsy sample, a multidisciplinary team discussion confirmed the diagnosis using the inclusion criteria applied in the INPLULSIS trial [14]. The clinical behavior of IPF patients included in the placebo arm of the INPULSIS trial was similar regardless the presence of a definite or possible UIP pattern on HRCT [15]. The HRCT scans were reported by the same radiologist to avoid interobserver variations in the characterization of the radiologic pattern. Pirfenidone was prescribed either under the manufacturer funded Named Patient Programme (NPP) or as a standard treatment approved by the Greek NHS.

Patients underwent pulmonary function tests (PFTs) including body plethysmography, spirometry and single breath test for determination of diffusing capacity of the lung for carbon monoxide (DLCO), at the time of IPF diagnosis and thereafter at 3, 6, 12, 24 and 36 months after the introduction of treatment. Blood tests for full blood count and liver and renal function obtained at the time of diagnosis and then once monthly for the first 6 months and subsequently once every 3 months. The clinical records of the patients were reviewed to assess the presence of comorbidities, treatment-related side-effects, compliance with the treatment and survival.

The survival in the UHH cohort was compared with the survival of a historic IPF cohort which included 212 patients referred to the Royal Brompton Hospital, London, UK (herein referred to as the RBH cohort) between December 1990 and December 1996 and has been previously described [13]. Acknowledging that the inclusion and diagnostic criteria for IPF differ between the UHH and the RBH cohort, we applied the criteria used in the UHH cohort to the RBH cohort. The HRCT scans and the biopsy samples were reviewed again in the RBH MDT meeting. To avoid biases due to inclusion of more severe cases in the RBH cohort, we excluded severe cases defined as having a DLco< 30%. This threshold is the same that was used for patient’s recruitment in the ASCEND trial [6].

In order to address the possibility of an interaction between treatment effect and age (given the major age difference between the cohorts), a sub-analysis was performed in which there was 1:1 age matching between the cohorts, with 64 patient couples. Eleven elderly patients from the UHH cohort could not be age-matched with RBH patients.

In order to minimise outcome differences due to the shorter duration of follow-up in the UHH cohort, a sub-analysis was performed in which follow-up was ended at 40 months.

Informed consent was obtained from all patients included in the UHH cohort. The study was approved by the Ethics Committees of the University Hospital of Herakleion (IRB number: 17030). Due to the retrospective nature of the analysis of the RBH cohort^13^, ethics committee approval was not required.

### Statistical analysis

Results were analyzed using STATA. Data were presented as mean values ± standard deviation for continuous and frequency (percentage) for nominal variables. Kaplan-Meier analysis and Cox proportional Hazards regression were used for survival analysis. A value of *p* < 0.05 was considered as statistically significant.

## Results

### Demographic characteristics of the UHH cohort

The demographic characteristics of the UHH cohort are shown in Table 1. The mean age was 74.9. Fifty-three patients (64.7%) were current or ex-smokers with a smoking history of 43.5 pack years. Mean FVC was 81.5% and mean DLco was 54.4% predicted. Seven patients (8.5%) had advanced disease. The radiologic pattern of definite UIP on HRCT was seen in 47 patients (57.3%) and the pattern of possible UIP was seen in 35 (42.7%). Thirteen patients underwent a surgical lung biopsy and the multidisciplinary team (MDT) discussion confirmed the diagnosis of IPF in the rest of the cases. Fifteen patients died during the study period. The main comorbidities are shown in Table 2. Comorbidities were either reported by the patient during the initial assessment as a new case or were diagnosed during the initial diagnostic work-up. Systemic hypertension was the most common comorbidity (64%), followed by gastroesophageal reflux disease (47%), ischemic heart disease (30%), heart failure (28%), combined pulmonary fibrosis and emphysema (21%), pulmonary hypertension (13%) and lung cancer (2%).

**Table 1:** Demographic characteristics of the UHH cohort (*n* = 82)

Age	74.9 ± 11^a^
Never smokers	29 (35.3%)
Smokers	53 (64.7%)
Pack Years	43.5 ± 32.1^a^
Radiological pattern (Definite UIP/Possible UIP)	47(57.3%)/35(42.7%)
Advanced disease (DLco< 30%)	7(8.5%)
Surgical lung biopsy	13 (15.9%)
FEV_1_%	87.4 ± 22.7^a^
FVC %	81.5 ± 19.5^a^
DLco %	54.4 ± 17.9^a^
Median follow-up time (IQR)	17.4 months (9.5–30.9).
Deaths	15 (18.3%)

*UIP* usual interstitial pneumonia, *FEV*_*1*_ forced expiratory volume in 1 s, *FVC* forced vital capacity, *DLco* diffusing capacity of the lung for carbon monoxide

^a^The values are expressed as mean ± SD

**Table 2:** Comorbidities in the UHH cohort

Comorbidities	*N* = 82
Systemic Hypertension	58 (64%)
Gastroesophageal Reflux Disease	42 (47%)
Ischemic Heart Disease	27 (30%)
Heart Failure	25 (28%)
Diabetes Mellitus	24 (27%)
Obstructive Sleep Apnea-Hypopnea Syndrome	23 (26%)
Depression	21 (23%)
Emphysema	19 (21%)
Pulmonary Hypertension	12 (13%)
Lung Cancer	2 (2%)
Venous Thromboembolism	1 (1%)

### Pirfenidone efficacy

#### Survival

**Fig. 1:**
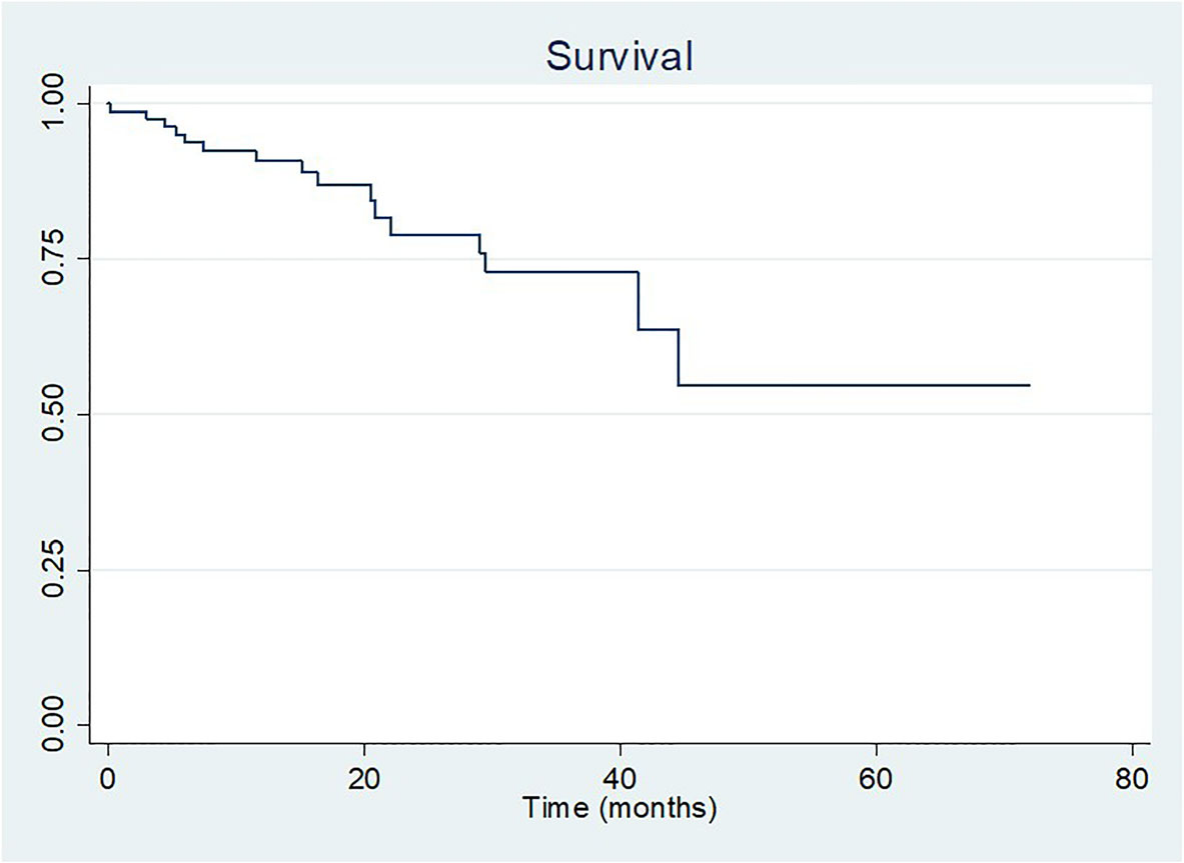
Kaplan Meier survival curve for the whole UHH cohort

Survival analysis with Kaplan Meier curve is shown in Fig. 1. In this intention to treat study, the 3-years survival in the UHH cohort was 73%. When we excluded the patients who discontinued the treatment (*n* = 22) because of side-effects or lack of adherence to treatment as it happens in the treatment arms of the phase III trials, the result did not change.

**Fig. 2:**
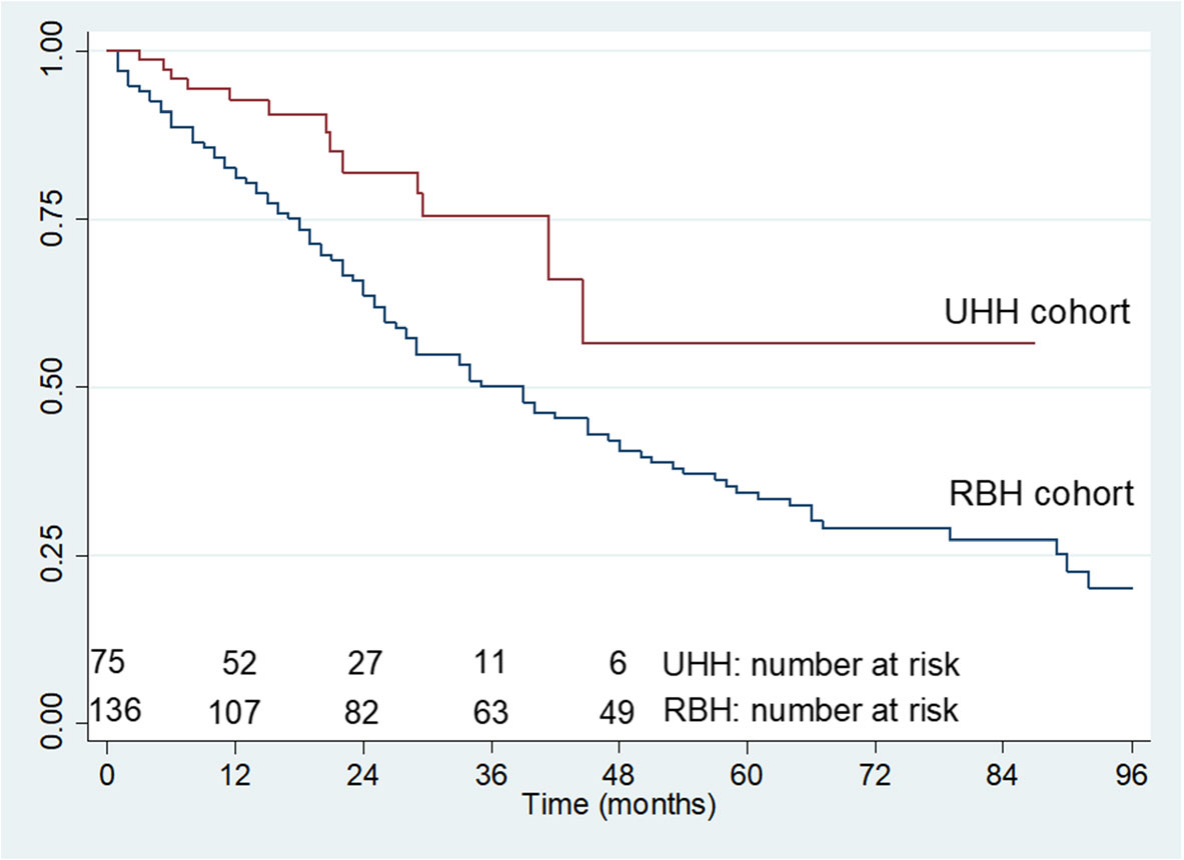
Kaplan Meier survival curve after adjustment for FVC, age and gender

In order to better understand the effect of Pirfenidone on survival, we compared the survival data from the UHH cohort to those obtained from a well characterized IPF cohort from a tertiary referral center in UK (Royal Brompton Hospital). To avoid biases due to the inclusion of more severe cases in the RBH cohort, we excluded cases with a DLco< 30%. The final analysis included 136 patients from the RBH cohort and 75 patients from the UHH cohort. In order to avoid using two cohorts in which different criteria for the diagnosis of IPF were used, we applied the same criteria used in UHH cohort [10, 14] to the RBH cohort. The characteristics of both cohorts are shown in Table 3. In unadjusted analysis, the survival in the UHH cohort was better than in the RBH cohort (HR:0.32, 95% CI: 0.19–0.53, *p* < 0.0001). After adjustment for FVC, age and gender the result did not change (HR:0.28, 95% CI: 0.16–0.48, *p* < 0.0001 (Table 4 and Fig. 2).

**Table 3:** Demographic characteristics of the patients from the UHH and RBH cohort included in the survival analysis. Seven patients with DLco< 30 were excluded from the initial UHH cohort

Patients data	UHH (*n* = 75)	RBH (*n* = 136)	*P* value
Age	72.5 ± 7.6^a^	62.5 ± 9.9^a^	*P* < 0.0001
Gender (male/female)	64/11	96/40	*P* < 0.01
Smoking status			
Never smokers	26	27	*P* = 0.08
Smokers	49	109	*P* = 0.34
Radiological pattern (Definite UIP/Possible UIP)	41/34	56/55	*P* = 0.7
Biopsy proven UIP	9	25	
FEV_1_	87.4 ± 21.5^a^	78.9 ± 15.6^a^	*P* < 0.0006
FVC	81.8 ± 17.8^a^	79.3 ± 18.0^a^	*P* = 0.16
DLco	56.7 ± 16.0^a^	46 ± 12.4^a^	*P* < 0.0001

*UIP* usual interstitial pneumonia, *FEV*_*1*_ forced expiratory volume in 1 s, *FVC* forced vital capacity, *DLco* diffusing capacity of the lung for carbon monoxide

^a^The values are expressed as mean ± SD

**Table 4:** Survival analysis. Proportional hazards model containing FVC, age and gender as covariates

	HR	95% CI	*P* value
Centre	0.28	0.16, 0.48	*P*<0.0001
Gender	1.96	1.19, 3.22	*P*<0.008
Age	1.03	1.01, 1.05	*P*<0.002
FVC	0.97	0.96, 0.98	*P*<0.0001

In an age-matched model comparing survival between cohorts in 64 age-matched patient couples, mortality was reduced in the pirfenidone treated cohort (HR 0.30, 95% CI 0.15, 0.59; *p* < 0.001. This finding was robust in a proportional hazards model, also containing FVC, age and gender as covariates (Table 5).

**Table 5:** Age-matched model comparing survival between cohorts in 64 age-matched patient couples

	HR	95% CI	*P* value
Centre	0.27	0.14, 0.55	*P* < 0.0001
Gender	1.96	0.99, 3.88	*P* < 0.053
Age	1.00	0.96, 1.04	*P* < 0.838
FVC	0.98	0.97, 1.00	*P* < 0.204

In a sub-analysis in which follow-up was terminated at 40 months, mortality was reduced in the pirfenidone-treated cohort (HR 0.40; 95%CI 0.21, 0.75; *p* < 0.005). This finding was robust in a proportional hazards model, also containing FVC, age and gender as covariates (Table 6).

**Table 6:** Survival sub-analysis at 40 months

	HR	95% CI	*P* value
Centre	0.28	0.14,0.54	*P* < 0.0001
Gender	1.72	0.94,3.16	*P* < 0.077
Age	1.02	1.00,1.05	*P* < 0.033
FVC	0.97	0.96,0.98	*P* < 0.0001

#### Safety

In the UHH cohort, 30 patients (33,3%) reported gastrointestinal discomfort and 17 patients (18,8%) have experienced photosensitivity. Finally, 3 patients (3.3%) discontinued treatment due to gastrointestinal adverse events, 9 (10%) patients discontinued due to photosensitivity and 10 (11.1%) due to non-compliance to treatment. The long-term safety results from this study are consistent with the known safety profile of pirfenidone, with no new safety signals observed.

## Discussion

This is a single-center observational, intention to treat study of efficacy and safety of Pirfenidone in IPF. We observed that treatment adherent patients had an increased 3 years survival rate of 73% despite the inclusion of patients who would normally be excluded from pharmaceutical trials. The approval of the antifibrotic agents has marked the end of the placebo-controlled studies in IPF. Nowadays, it is not ethical to withhold Pirfenidone therapy to establish a true control group and therefore we chose to use a historical cohort as a control group. Importantly, the use of Pirfenidone was associated with a significantly higher survival compared to a historical cohort studied in the pre-pirfenidone era after adjustment for demographic characteristics and baseline disease severity as judged by FVC. When we examined age and follow-up time between the two cohorts as confounding factors, the effect of Pirfenidone on survival remained robust. We chose to use survival as a “response measure” instead of long-term pulmonary function trends which are seriously biased by the fact that they can only be evaluated in survivors. This is a major problem in open-label studies and has been explored in a recent commentary in Lancet Respiratory Medicine [16]. The safety profile was comparable to that reported in phase III and extension trial analysis [6, 9].

It is well known that phase III IPF trials have not been designed to estimate long-term survival. Designing and conducting an adequately powered survival study in IPF is challenging due to the rare nature of the disease and the need for extended follow-up. Open-label extension studies in patients with IPF who completed the phase III trials are expected to provide answers on the survival benefit. However, patients participating in randomized controlled trials (RCTs) represent a highly selected group of patients fulfilling strict inclusion and exclusion criteria as shown by the high screen failure of 70% in the ASCEND study [6]. Real world observational studies could provide evidence regarding the efficacy of the antifibrotic compounds although patients are often different than those enrolled in clinical trials for the presence of comorbidities and for advanced disease at presentation as judged by pre-treatment FVC and DLco values. In the UHH cohort, seven patients with advanced disease (DLCO< 30%) and a significant number of patients with more than one comorbidities such as emphysema, ischemic heart disease and diabetes were included. Therefore, a significant number of our real-world patients would not fulfil the inclusion criteria for clinical trials.

Despite the inclusion of these patients, we still observed a relatively high 3-years survival rate. When we excluded from the analysis the patients who discontinued the treatment due to side effects or lack of adherence, the survival benefit remained unchanged. To strengthen our results, we compared the survival in our intention to treat cohort to a non-intention to treat historical cohort. We found that, in unadjusted analysis, the UHH cohort had a 30% increased survival and the survival benefit remained significant after adjustment for age, gender and baseline severity. In order to exclude the likelihood that the observed survival benefit was due to the inclusion of less cases with advanced disease in the UHH cohort, we repeated the analysis after excluding patients with a DLco< 30%. The results did not change. Plainly, we cannot exclude whether the observed benefit was due to the adverse effect of the immunosuppressive treatment used as standard IPF therapy in the pre-antifibrotic era. It is now well known that the combination of low dose of prednisolone with azathioprine and N-acetyl-cysteine has been proved not efficacious and deleterious in IPF [17]. However, it is also acknowledged that the increased number of deaths and hospitalizations due to infections was observed when the patients were receiving significantly higher doses of prednisolone [17]. According to the therapeutic algorithm of the RBH hospital, doses of prednisolone higher than 10 mg were not used when the RBH cohort was studied. Keeping this in mind, we can conclude that not using immunosuppression and using pirfenidone instead, provides a 30% survival benefit in IPF. A recent study confirmed that the use of pirfenidone is associated with a survival benefit. The authors observed that pirfenidone treated patients have a 2.47 years longer life expectancy than IPF patients receiving best supportive care [18]. Best supportive care includes interventions such as pulmonary rehabilitation, supplemental oxygen therapy, and/or other symptomatic treatments, which do not have any effect on IPF survival.

Our finding is important because the 3 years survival rate did not change regardless the exclusion of the cases, which discontinued Pirfenidone. In our survival analysis, we included all patients who received Pirfenidone at baseline, even those who interrupted the treatment for different reasons. In the open-label extension RECAP study the median on-treatment survival from the first dose of 2403 mg/day pirfenidone was 77.2 months [9]. However, patients who interrupted the treatment were not included in this analysis and as observed in trials outside IPF, withdrawals from drug trials have worse outcome [19–21]. Therefore, the open-label data do not actually show true mortality outcome on intention to treat with Pirfenidone.

In the UHH cohort, 30 patients reported gastrointestinal discomfort and 17 patients have experienced photosensitivity. Three patients discontinued treatment due to gastrointestinal adverse events, 9 due to photosensitivity and 10 due to non-compliance. The rate of discontinuation due to adverse effects is 13.3% which is similar to the rate observed in the ASCEND and CAPACITY trials (14.4% and 15 respectively in the treatment arms) [6, 22]. One of the most frequent adverse events was a photosensitivity reaction, which is totally expected keeping in mind the weather condition in south Greece. The rate of discontinuation due to a photosensitivity reaction is lower than in phase III trials and recently published real-life studies [6, 22–25]. This is mainly due to a very detailed discussion between the treating physicians and the patient prior to the introduction of treatment. A leaflet with helpful recommendations was given to every patient as a skin protection guide. The use of wide-brimmed hats, sunglasses, long-sleeved shirts, trousers, gloves if possible, and sunscreens with high sun protection factor (ie, > 50) with UV-A/UV-B protection is recommended [4]. Real-life studies have highlighted the importance of a frequent review of the patients by a specialist nurse as well as the importance of a contact number given to the patients so that they can communicate whenever they have questions about the drug or when they experience side effects. In UHH, a dedicated research fellow provides regular specialist input to provide support and education in order to improve concordance to treatment.

## Conclusion

To the best of our knowledge, this is the first intention to treat real-life study showing an increased 3 years survival rate in patients treated with Pirfenidone and a survival benefit of 30% compared to patients treated with no antifibrotic agents. The effect of Pirfenidone on survival is remarkable if ones takes into account that patients with comorbidities and severe disease have been included in the UHH cohort unlike what happens in pharmaceutical trials. We confirmed the safety profile of Pirfenidone and observed a lower rate of discontinuation due to photosensitivity reactions and stressed the importance of patient’s counselling before the initiation of treatment. More real life studies with a higher number of patients who are unlikely to be eligible for inclusion in pharmaceutical trials are needed to evaluate the effect of Pirfenidone on disease progression.

## Abbreviations

DL_CO_: Diffusing capacity for carbon monoxide
FEV_1_: Forced expiratory volume in 1 s
FVC: Forced vital capacity
HR: Hazard ratio
HRCT: High resolution computed tomography
IPF: Idiopathic pulmonary fibrosis
MDT: Multidisciplinary team
NPP: Named Patient Program
PFTs: Pulmonary function tests
RBH: Royal Brompton Hospital
RCTs: Randomized controlled trials
UHH: University Hospital of Herakleion
UIP: Usual interstitial pneumonia
